# Imaging the antimicrobial mechanism(s) of cathelicidin-2

**DOI:** 10.1038/srep32948

**Published:** 2016-09-14

**Authors:** Viktoria A. F. Schneider, Maarten Coorens, Soledad R. Ordonez, Johanna L. M. Tjeerdsma-van Bokhoven, George Posthuma, Albert van Dijk, Henk P. Haagsman, Edwin J. A. Veldhuizen

**Affiliations:** 1 Department of Infectious Diseases and Immunology, Division Molecular Host Defence, Faculty of Veterinary Medicine, Utrecht University, Utrecht, The Netherlands; 2 Department of Cell Biology, Cell Microscopy Core, University Medical Centre Utrecht, Utrecht, The Netherlands

## Abstract

Host defence peptides (HDPs) have the potential to become alternatives to conventional antibiotics in human and veterinary medicine. The HDP chicken cathelicidin-2 (CATH-2) has immunomodulatory and direct killing activities at micromolar concentrations. In this study the mechanism of action of CATH-2 against *Escherichia coli* (*E. coli*) was investigated in great detail using a unique combination of imaging and biophysical techniques. Live-imaging with confocal fluorescence microscopy demonstrated that FITC-labelled CATH-2 mainly localized at the membrane of *E. coli*. Upon binding, the bacterial membrane was readily permeabilized as was shown by propidium iodide influx into the cell. Concentration- and time-dependent effects of the peptide on *E. coli* cells were examined by transmission electron microscopy (TEM). CATH-2 treatment was found to induce dose-dependent morphological changes in *E. coli*. At sub-minimal inhibitory concentrations (sub-MIC), intracellular granulation, enhanced vesicle release and wrinkled membranes were observed, while membrane breakage and cell lysis occurred at MIC values. These effects were visible within 1–5 minute of peptide exposure. Immuno-gold TEM showed CATH-2 binding to bacterial membranes. At sub-MIC values the peptide rapidly localized intracellularly without visible membrane permeabilization. It is concluded that CATH-2 has detrimental effects on *E. coli* at concentrations that do not immediately kill the bacteria.

Since the 1980s the number of antibiotic resistant infections has consistently increased in both humans and animals^1^,^2^. To overcome this world-wide problem alternative treatments are required. Attractive alternatives to conventional antibiotics are host defence peptides (HDP)^3^. Chicken cathelicidin-2 (CATH-2), one of the four known chicken cathelicidin-like HDPs, is an arginine-lysine rich peptide with both immunomodulatory and strong broad-spectrum antibacterial activity, which is not inactivated by serum or high salt concentrations^4^,^5^,^6^. CATH-2 consists of two alpha-helical segments separated by a proline-induced kink, which is essential for the direct killing activity of the peptide^7^. Previous studies have already shown that CATH-2 exhibits broad-range bactericidal activity, however its antibacterial mode of action is still unrevealed.

The net positive charge and amphipathicity of antimicrobial HDPs enable a strong interaction with negatively charged outer (lipopolysaccharide; LPS) and inner (phospholipids) membranes of Gram-negative bacteria. This interaction can lead to destabilization and permeabilization of bacterial membranes, for which, depending on the exact interaction and peptide, several different models have been described. However, besides the described effects on the membrane, several studies have shown that peptides can actually cross membranes without membrane damage and reach intracellular targets such as ribosomes, DNA or other intracellular molecules. This can subsequently result in, among others, inhibition of DNA or RNA synthesis, protein synthesis, or protein folding. For extensive reviews on the multiple modes of action of HDPs see: Brogden and Nguyen, Haney and Vogel^8^,^9^.

The aim of this study was to investigate the antibacterial killing mechanism of CATH-2. To achieve this, a unique combination of imaging techniques and binding assays was used during this study. Live-imaging fluorescence microscopy demonstrated the real-time attack of CATH-2 on *E. coli*. Morphological changes of the bacteria after peptide treatment were determined with transmission electron microscopy and immunogold electron microscopy was performed in order to determine peptide localization. Lastly, isothermal titration calorimetry and competition assays were used to study the peptide binding with LPS. Our results show that the bacterial membrane is an important (initial) target of CATH-2, but also that CATH-2 is present intracellularly at sub-MIC levels and has visible detrimental effects on bacterial cell structure.

## Results

### CATH-2 rapidly kills bacteria at micromolar concentrations

**Figure 1:**
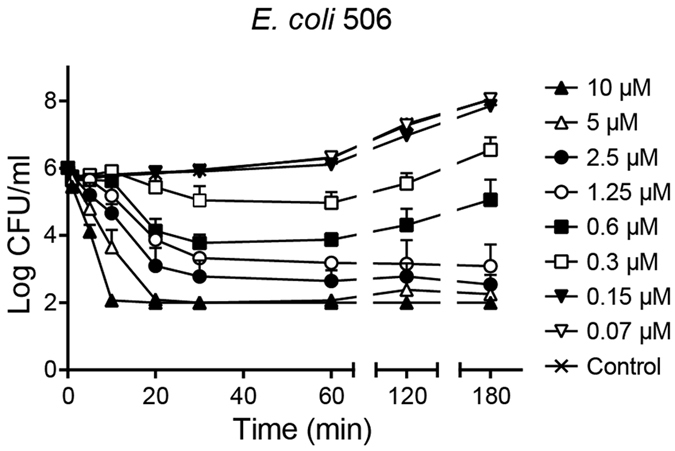
CATH-2 rapidly kills *E. coli*. *E. coli*506 was co-incubated with different CATH-2 concentrations at 37 °C for different time points (1, 5, 10, 30, 60, 120 and 180 min). Each mixture was aliquoted at the various time intervals, serially diluted and spread plated on TSA plates. After 16 h at 37 °C plates were counted for surviving bacteria. Data represent three independent measurements (means ± SEM).

CATH-2 and FITC-labelled CATH-2 were tested for their antibacterial activity using colony count assays. No differences in the minimal inhibitory concentration (MIC) values of the two peptides against *E. coli* were observed. Both peptides showed a MIC value of 5–10 μM. In addition, the killing kinetics of CATH-2 against *E. coli* was tested in order to detect the speed of killing at different peptide concentrations. At MIC level (10 μM) CATH-2 killed the bacteria within 10 min. At low peptide concentrations an initial decrease in the number of surviving bacterial cells was observed, however after 60 minutes surviving bacteria recuperated from the peptide attack and started growing again (Fig. 1).

### CATH-2 binds and permeabilizes the bacterial membrane

**Figure 2:**
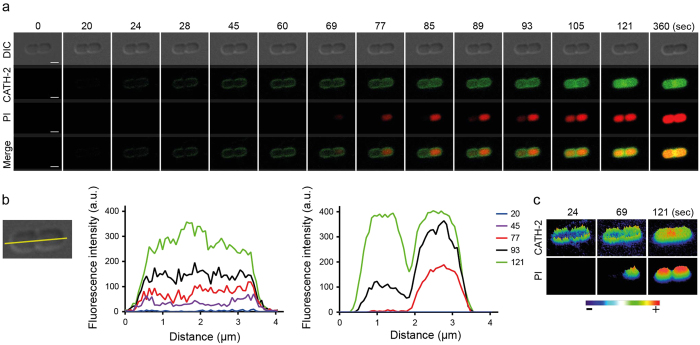
Snapshots of a single cell show the fast membrane binding and permeabilization of CATH-2. Various time lapses within a 6 min period are shown (**a**). Additionally, the fluorescent intensity of FITC-CATH-2 (0.9 μM) and PI (5.1 μM) was measured in both parts of a dividing bacterium (*E. coli* 506), based on transverse linescans (yellow line) (**b**). Heat intensity plots show the specific binding sites of FITC-CATH-2 and PI (**c**). Bars, 1 μm.

Time-lapse imaging was performed using FITC-labelled CATH-2 and the permeability marker propidium iodide (PI). Instantly after adding the peptide, CATH-2 localized to the membrane of *E. coli* 506 and was shortly after also detectable in the cytoplasm. Propidium iodide was also detectable indicating that the bacterial membrane is permeabilized (Fig. 2a, Supplementary Movie 1). More detailed analysis of the images using transverse linescans on the horizontal axis of the shown bacteria, indicated that CATH-2 can be detected as early as t = 45 sec in the cytoplasm of the bacteria, while the first PI detection was observed after 93 sec (Fig. 2b). This indicates that the peptide translocates over the membrane into the cytoplasm before the membrane is actually permeabilized. This order of events was seen with small variations, in all bacterial cells studied (Supplementary Movie S2). Heat intensity plots confirmed the rapid membrane binding of CATH-2, especially at the bacterial septum of dividing cells, higher intensities levels were observed (Fig. 2c).

### CATH-2 induces dose-dependent morphological changes of *E. coli*

**Figure 3:**
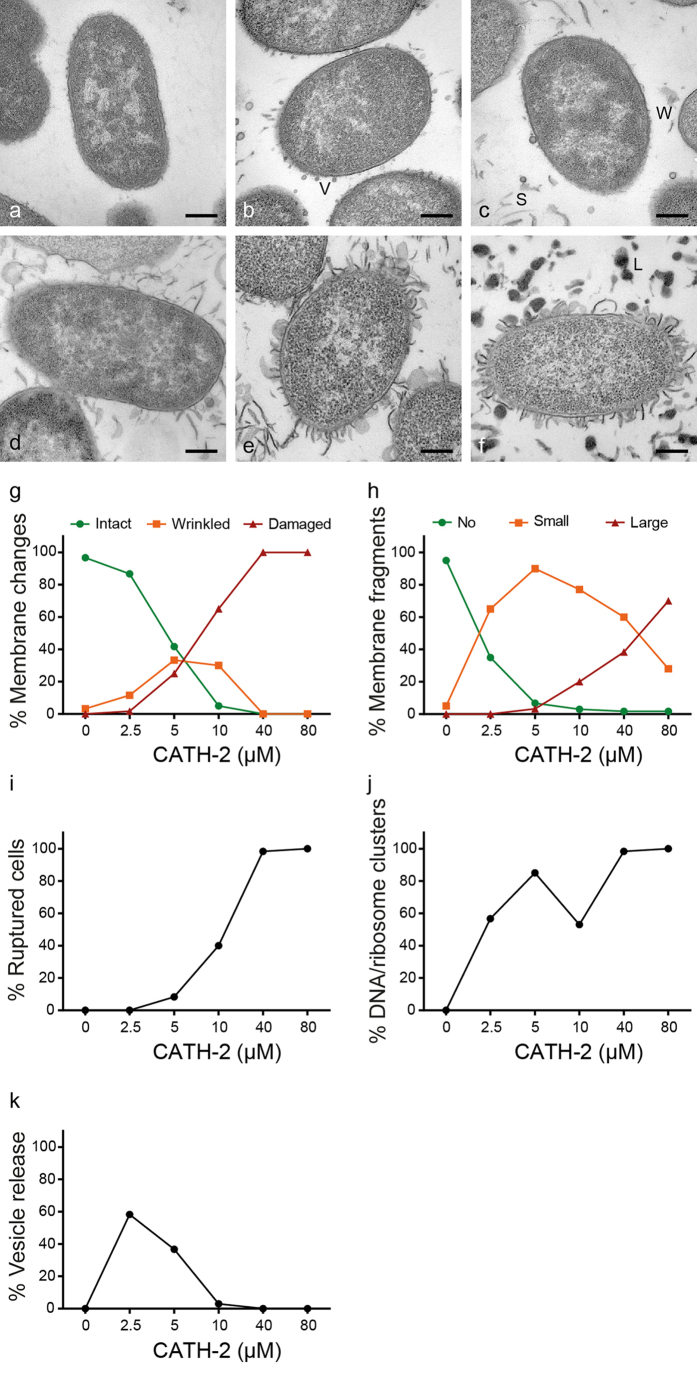
CATH-2 induced morphological changes of *E. coli* 506 determined by TEM. Representative images are shown for 0 μM (**a**), 2.5 μM (**b**), 5 μM (**c**), 10 μM (**d**), 40 μM (**e**) and 80 μM (**f**). In total 60 cells of two independent experiments were analysed per peptide concentration. V, vesicles; W, wrinkled membranes; S, small membrane fragments; L, large membrane fragments. Morphological changes were quantified in membrane intactness (**g**) and dissociated membrane fragments in the section (**h**). Additionally, morphological changes were classified in percentage of ruptured cells (**i**), DNA and ribosome clusters (**j**) and small vesicle release (**k**). Bars, 200 nm.

Morphological changes of *E. coli* 506 cells after a 30 min treatment with different peptide concentrations were determined using transmission electron microscopy (TEM). Overall, non-peptide treated bacteria had intact membranes and an evenly intracellular distribution of DNA and ribosomes rich area’s (Fig. 3; light and darker area’s respectively). At low peptide concentrations (2.5 and 5 μM, 1/16 and 1/8 MIC at the bacterial density used, respectively) CATH-2 exposure resulted in wrinkling of bacterial membranes and to some extent dissociation of membrane fragments. At 10 μM CATH-2 exposure induced membrane damage and strongly enhanced the amount of dissociated membrane fragments and number of ruptured cells. Interestingly, a marked release of small vesicles from the membrane was clearly visible in the presence of 2.5 μM CATH-2. At higher peptide concentrations no vesicle release was observed. At the same concentration intracellular effects were observed, as the DNA started to cluster in the centre of the cell and ribosomes were directed towards the inner membrane of the bacteria (Fig. 3a–f).

Quantification of these effects showed that 5 μM CATH-2 caused strong membrane effects, i.e. almost 30% of the cells had wrinkled membranes and in 25% of the cells membrane ruptures were observed (Fig. 3g). Increasing the peptide concentration doubled the number of damaged membranes, enhanced the number of dissociated membrane parts in the section and ruptured cells (Fig. 3g–i). Interestingly, at the lowest peptide concentration used 60% of the cells showed redistribution of DNA and ribosomes and a 60% increase in vesicle release was observed (Fig. 3j,k). These results suggest that besides direct effects on the bacterial membrane, CATH-2 mediates intracellular effects, either directly through small amounts of translocated peptide or indirectly by the membrane-bound CATH-2. Similar results were observed with negative-staining TEM (Supplementary Fig. S1a–e). At sub-MIC formation of small bleb-like structures was observed, which was reduced at higher CATH-2 concentrations (Supplementary Fig. S1f). At 10 and 40 μM complete membrane disruptions were observed (Supplementary Fig. S1g). Additionally with ascending concentrations the flagella of *E. coli* cells disappeared (Supplementary Fig. S1h).

**Figure 4:**
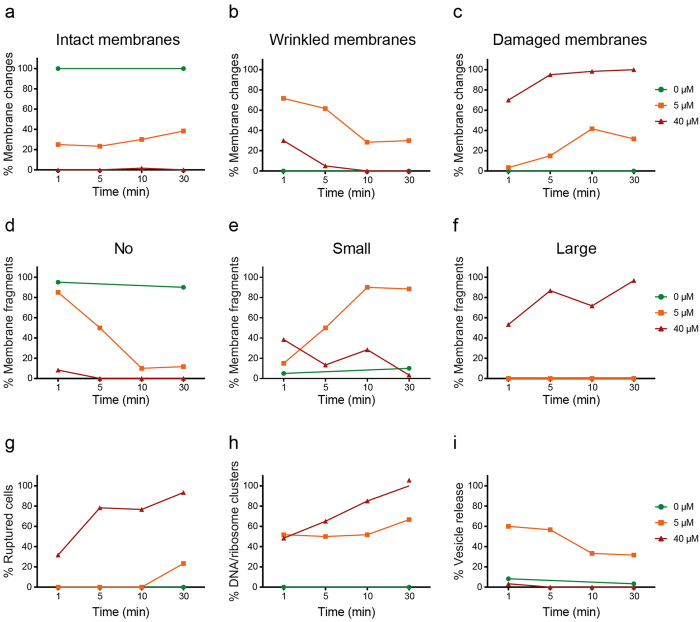
Time dependent CATH-2 induced morphological changes of *E. coli* 506. Morphological changes were determined of two independent experiments by TEM. Sixty cells per condition were analysed. Membranes were classified as intact (**a**), wrinkled (**b**) or damaged (**c**). Dissociated membrane fragments were identified as no (**d**), small (**e**) or large (**f**). Additionally, ruptured cells (**g**), DNA and ribosome clusters (**h**) and small vesicle release (**i**) were determined based on qualitative observations.

To gain further insight in the order of events and the actual speed at which CATH-2 induced its effects, *E. coli* exposure to CATH-2 was examined at different time points (Fig. 4a–i and Supplementary Fig. S2). A one-minute exposure of bacteria to 5 μM peptide resulted in wrinkled bacterial membranes. The number of wrinkled membranes per cell decreased with longer peptide incubation times, whereas simultaneously the number of damaged membranes and dissociated membrane fragments in the sections increased. Furthermore, DNA and ribosome redistribution and high vesicle release was observed after short (1–5 min) peptide incubation times. At the highest CATH-2 concentration tested, tremendous effects on the bacterial membranes were observed. After incubating the bacteria for one minute with 40 μM CATH-2 almost 70% of the cells had damaged membranes and in more than half of the analysed sections, large dissociated membrane fragments were observed. These numbers consistently increased at later time points. To conclude, these results showed that prominent effects on the membrane and also inside the cell are visible after very short incubation times (1–5 min) even at low peptide concentrations.

### CATH-2 localizes intracellularly at sub-MIC levels

**Figure 5:**
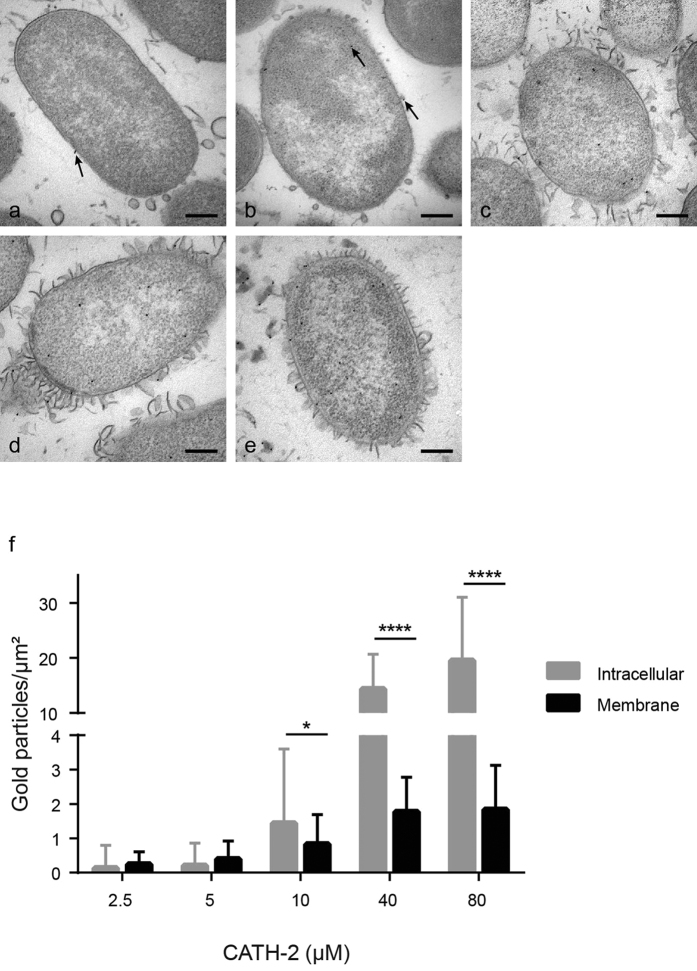
Localization of CATH-2 determined with immuno-gold labelling and quantification of gold particles. For each peptide concentration a representative TEM image is shown: 2.5 μM (**a**), 5 μM (**b**), 10 μM (**c**), 40 μM (**d**) and 80 μM (**e**). The number of gold particles (black arrow) per μm^2^ is divided in intracellular (grey bar) and membrane (black bar) (**f**). (means ± SEM) *P < 0.05 and ****P < 0.0001 for intracellular vs membrane. All peptide-treated samples were corrected for background staining. Bars, 200 nm.

To examine if CATH-2 translocates after adherence to the bacterial surface, TEM was combined with immunogold-labelling. Bacteria incubated for 30 min with 2.5 μM CATH-2, mostly showed localization of the peptide at the membrane. At 5 μM CATH-2 penetrated the bacterial membrane and CATH-2 was also found in the cytoplasm. At higher CATH-2 concentrations increasing numbers of randomly distributed gold particles in the bacteria and on the bacterial membrane were observed (Fig. 5a,b,f). Compared to 5 μM, incubation of bacteria with 10 μM peptide resulted in an increased (7-fold; 1.44 gold particles/μm^2^) number of intracellular electron-dense immunogold complexes and gold particles localized at the membrane (2-fold; 0.83 gold particles/μm^2^). At 40 μM (MIC) and 80 μM, high numbers of gold particles (>14 gold particles/μm^2^) within the cells were seen (Fig. 5c–f).

**Figure 6:**
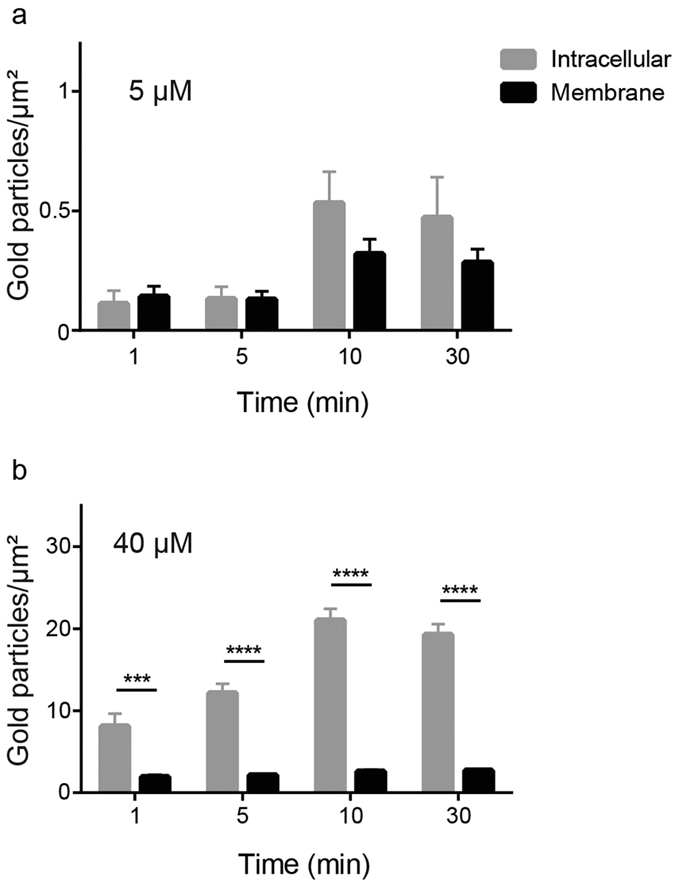
Time dependent CATH-2 localization on *E. coli* 506. Electron-dense immunogold complexes were counted in two different concentrations, 5 μM (**a**) and 40 μM (**b**), and separated in intracellular (grey bar) and membrane (black bar). (means ± SEM) ****P < 0.0001 and ***P < 0.001 for intracellular vs membrane. All peptide-treated samples were corrected for background staining.

Furthermore, the kinetics of CATH-2 localization at two different peptide concentrations was studied. After a 5 min bacterial exposure to 5 μM peptide, CATH-2 was observed equally at the bacterial membrane or intracellularly, while at later time points (10 and 30 min) the number of intracellular electron-dense immunogold particles was strongly increased (Fig. 6a and Supplementary Fig. S3). At 40 μM, the concentration where the TEM experiments showed very rapid disruption of the bacterial membrane, immuno-electron microscopy showed that CATH-2 immediately enters the cell after a one-minute exposure time (Fig. 6b and Supplementary Fig. S3). Longer incubation time did not further affect the CATH-2 localization indicating that at MIC values CATH-2 effects are almost instantaneous. To conclude, the TEM and immuno-TEM data combined showed that at sub-MIC level CATH-2 initially binds the bacterial membrane and then translocates intracellularly within a few minutes. This is accompanied by intracellular morphological changes of the bacterial cell as observed by redistribution of DNA and ribosomes. However, at MIC values these separate steps cannot be distinguished anymore and the observed morphological changes and CATH-2 localization seem almost immediate.

### CATH-2 binds to LPS

Since the initial interaction between CATH-2 and *E. coli* is localized to the bacterial membrane, it is likely that LPS plays an important role in the early peptide localization. To determine whether differences in LPS types have an effect on the activity of CATH-2, antimicrobial activity tests of CATH-2 against several rough and smooth LPS containing *E. coli* strains were performed. MIC values of CATH-2 against *E. coli* ATCC 25922 (smooth LPS) were 2–8 fold higher than rough LPS containing *E. coli* K12 strains, suggesting that CATH-2 binds to the O-antigen part of LPS (Table 1).

**Figure 7:**
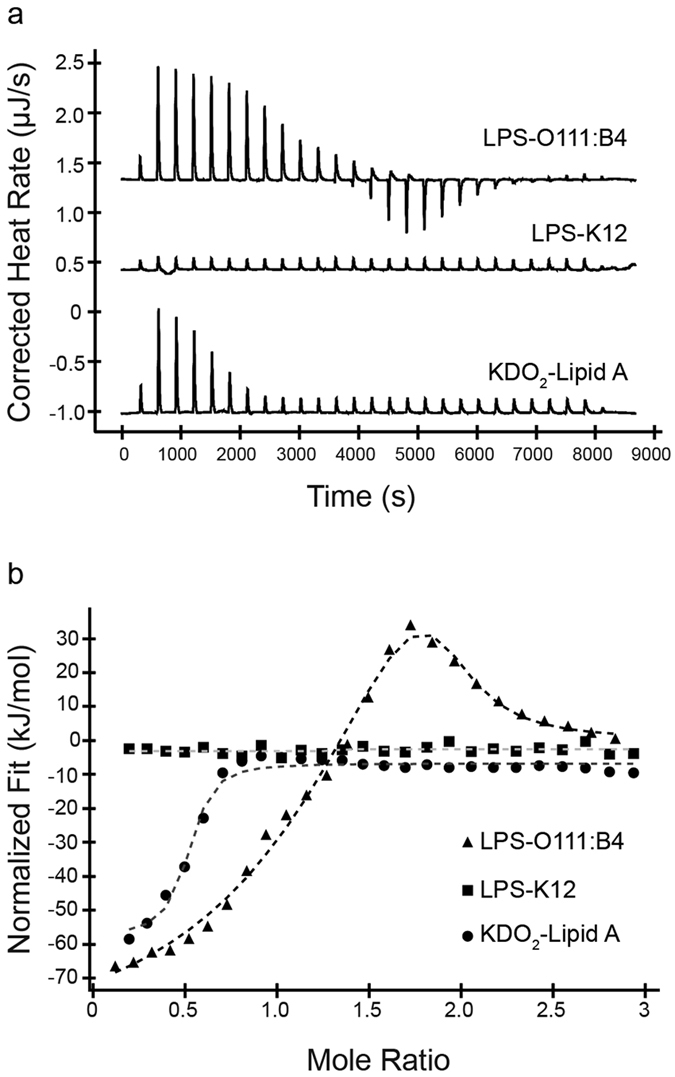
CATH-2 interaction with different LPS compounds. Thermograms of ITC experiments with titrations of CATH-2 into LPS-O111:B4, LPS-K12 and KDO_2_-Lipid A (**a**). Released heat versus molar ratio of CATH-2 and LPS compounds (**b**).

To determine whether differences in interaction between CATH-2 and LPS could explain the difference in killing efficiency of rough vs. smooth *E. coli*, isothermal titration calorimetry (ITC) experiments and competition assays were performed. ITC showed clear interaction between CATH-2 and smooth LPS, with an initial exothermic interaction followed by an endothermic one. Interestingly, titrations with CATH-2 into a rough LPS solution showed no indication of binding, while CATH-2 was able to interact with the even shorter Lipid A (Fig. 7a,b). Competition assays confirmed our findings, showing that the antibacterial activity of the peptide was inhibited by prior binding to smooth LPS or Lipid A but not to rough LPS (Supplementary Fig. S4a–c).

Finally, live-imaging studies were performed on *E. coli* K12, similar to the studies shown in Fig. 2. These experiments showed that the peptide is not binding to the membrane as observed for *E. coli* 506, yet causes membrane permeabilization and PI entrance (Supplementary Fig. S5).

## Discussion

In this study the antibacterial mechanism of action of CATH-2 was investigated using various imaging techniques involving confocal live-imaging and (immuno-gold) transmission electron microscopy. This combination of techniques, which has not been used before for mechanistic studies of antimicrobial peptides, provides a clear insight into the real-time localization and mechanism of action of CATH-2 at (sub-)MIC values.

CATH-2 was shown to have quick killing kinetics as within 10 minutes bacterial killing was observed. Several studies have already shown that the bactericidal activity of peptides is usually within minutes of the first interaction with bacteria^10^. In addition, our study showed that lower peptide concentrations (1/16 MIC) result in reduced numbers of surviving bacteria, however, bacterial growth resumed after 60 minutes. These results indicated that the peptide is not bactericidal for the whole bacterial population, since surviving bacteria can overcome the peptide-induced damage and start growing again.

Confocal fluorescence microscopy showed that FITC-labelled CATH-2 initially mainly localizes at the membrane of *E. coli*, with a higher intensity of binding at the bacterial septum of dividing cells. This preferred localization of the peptide at the septum can be explained by the presence of a double membrane (and thus more binding area), but specific binding to proteins or lipids enriched in the bacterial septum cannot be excluded. Similar studies were performed with LL-37 and demonstrated that the peptide binds and permeabilizes the outer membrane first, resulting in release of periplasmic components, and then, after approximately 15–20 min reaches and permeabilizes the inner membrane of *E. coli*^11^. In our study, these separate steps, if present for CATH-2, could not be observed because CATH-2 seems to act much quicker with an inner membrane permeabilization of approximately one minute. Permeabilization of the bacterial membrane is a well-documented mechanism of action for many antimicrobial peptides. Several peptides, for example LL-37, magainin 2 and cecropin A, have shown to exhibit pore-forming activity by direct binding to the bacterial membrane, while other peptides permeabilize the membrane using a carpet model such as PMAP-23^12^,^13^,^14^,^15^,^16^. Time-lapse imaging does not allow to correlate peptide concentrations to sub-MIC or MIC values. Therefore, TEM was performed to study morphological changes of the cells after as a function of peptide concentration.

The TEM studies showed that, at MIC levels, bacteria were immediately disrupted and the peptide was localized both intracellularly and on the bacterial membranes. At this concentration (and speed of the killing process) no information about the order of events of CATH-2’s antimicrobial mechanism of action could be determined. However, at sub-MIC levels these steps are more easily visualized. At 1/8 MIC, intracellular localization and morphological changes including, wrinkling of the membranes, relocalization of DNA and ribosomes, and increased vesicle release were observed. Gold particles were initially detected on the bacterial membrane and after 10 and 30 min immunogold complexes were also observed inside the cells without a clear disruption of the bacterial membrane. Similar findings were demonstrated with the proline-rich peptide Bac7 based on membrane permeabilization and immuno-electron microscopy. Bac7 (below MIC level) was suggested to bind to an intracellular target after entering the cell in a stereospecific manner in the absence of pore formation or membrane permeabilization^17^. In fact, the whole family of proline-rich peptides, the cell penetrating peptide P7 and buforin II are thought to act intracellularly instead of working via a lytic mechanism of action^18^,^19^,^20^,^21^,^22^,^23^,^24^.

Our data indicated that CATH-2 quickly associates with the bacterial outer membrane, most likely with LPS. The observed LPS binding of CATH-2 was in line with previous studies demonstrating that CATH-2 neutralizes LPS induced cytokine production of PBMCs^5^. Recent studies on PMAP-23 have shown that at the MIC value of 10 μM the complete bacterial surface was covered by the peptide^25^. Considering the relatively similar peptide dimensions and MIC value for CATH-2 compared to PMAP-23 it is anticipated that indeed a near complete (LPS) saturation of the outer membrane seems to be required for antimicrobial activity. However, the binding of CATH-2 to LPS does not necessarily contribute to the antimicrobial mechanism. It is more likely that LPS in the outer membrane is a barrier that the peptide has to overcome to reach its target, either the cytoplasmic membrane or an intracellular target or both. Similar binding effects were described for the proline-rich peptides drosocin, pyrrhocoricin and apidaecin^24^. LPS is their initial (docking) target, which plays an important role in the cell-mediated entry of these peptides to finally initialize the intracellular killing cascade^22^,^24^,^26^.

Our results emphasize the importance of sub-MIC values in order to detect an antibacterial mode of action. In the case of CATH-2, at sub-MIC the peptide was not absorbed by LPS and showed membrane and intracellular changes, whereas at MIC values membrane leakage is observed after complete LPS saturation. Together, our current findings suggest that besides the membrane-permeabilizing effect of CATH-2, the peptide may trigger a killing cascade at sub-MIC values by escaping LPS. To date various antimicrobial peptides have been reported that exert a mechanism of action combining membrane and intracellular targeting^17^,^27^,^28^,^29^. Oligo-acyl-lysyl antimicrobial peptide C_12_K-2β_12_ and Bac7 were demonstrated to bind to intracellular target(s) at sub-MIC values and exhibited a membrane-attacking mode of action at higher peptide concentrations^17^,^28^. These studies, together with our results, indicate that CATH-2 might exert similar dual activity on both intracellular targets and the bacterial membrane.

In conclusion, this report provides a detailed overview on the antibacterial mode of action(s) of a chicken cathelicidin using different imaging and molecular interaction studies. Visualization of the real-time attack of the peptide and the determination of peptide localization and morphological changes of the bacteria have shed light on the mechanisms of action at MIC and sub-MIC values. Based on these findings, future research on the antibacterial killing of (new) antimicrobials should not only focus on MIC values, as these concentrations will not tell the complete killing story of an antibacterial drug.

## Material and Methods

### Peptide

CATH-2 was obtained from CPC Scientific Inc. (Sunnyvale, USA). Fluorescently labelled CATH-2 was made as described previously^30^.

### Bacterial strains and growth conditions

Six *Escherichia coli* (*E. coli*) strains were used in this study, i.e. *E. coli* ATCC 25922, ATCC 4157, K12, LMC500, MC4100 and 506 (O78K80), of which the latter was used as the reference strain in all experiments^31^,^32^. All bacterial strains were grown in Tryptone Soy Broth (TSB; Oxoid Limited, Hampshire, UK) and on Tryptone Soy Agar (TSA; Oxoid Ltd). For all experiments bacteria were inoculated and grown overnight in TSB at 37 °C. The next day bacteria were transferred to a fresh TSB tube and grown to mid-logarithmic phase.

### Colony count assay

To measure the antibacterial activity of CATH-2 with or without FITC label, colony count assays were performed as described before^33^. In short, mid-logarithmic phase cultures were washed once in TSB and diluted to 2 × 10^6 ^CFU/ml. Subsequently, bacteria were exposed to peptide (0–40 μM) for 3 h at 37 °C. Afterwards, the mixtures were diluted 50–5,000 fold, spread plated on TSA plates and after 16 h at 37 °C surviving colonies were counted.

### Killing kinetics

To assess the time point of bacterial growth inhibition, killing kinetics with CATH-2 were performed. Peptide concentrations ranging from 0–20 μM CATH-2 were incubated with mid-logarithmic *E. coli* (2 × 10^6 ^CFU/ml). At 1, 5, 10, 20, 30, 60, 120 and 180 min, 100 μl aliquots were taken and immediately plated on TSA. Additionally, 20 μl aliquots were diluted 10- to 1,000-fold and again 100 μl was plated. After 16 h incubation at 37 °C the surviving bacteria were counted.

### Confocal microscopy

To visualize peptide localization and permeability of the *E. coli* cytosolic membrane, time-lapse live-imaging was performed. Mid-logarithmic *E. coli* 506 was washed with 5 mM Hepes (pH 7.4, Sigma-Aldrich, Zwijndrecht, The Netherlands). Cells were pelleted and resuspended in 1 ml of Hepes buffer to obtain a high bacterial density. Silanized coverslips (Gerhard Menzel GmBH, Braunschweig, Germany) were prepared with 2% (3-Aminopropyl) triethoxysilane (SAA; Sigma-Aldrich) in acetone. Thirty microliters of 1% low-melting agarose (Agarose Type I low EEO, Sigma-Aldrich) was added in the center of the silanized coverslips. The same amount of bacteria was added and resuspended in the agarose. A second coverslip was placed on top. After 2 min the upper coverslip was removed and the remaining agarose layer was fixed in an Attofluor cell chamber and 900 μl HEPES buffer was added. At this point the permeability marker propidium iodide (PI; 5.1 μM final concentration; Sigma-Aldrich) was added. After focusing the cells, the movie was started and 60 μl 20 μM FITC-labelled CATH-2 (0.9 μM final concentration) was added to the coverslip. Experiments were performed at the Leica SPE-II and Nikon A1R at the Centre for Cell Imaging (CCI) at the faculty of Veterinary Medicine in Utrecht. FITC-CATH-2 and PI were detected with a Sapphire Blue Coherent laser (488 nm) and a Sapphire Yellow Coherent laser (561 nm). For data analysis ImageJ/FIJI software and NIS-Elements of Nikon was used.

### Transmission electron microscopy

Concentration and time dependent bactericidal effects of CATH-2 were further investigated by performing transmission electron microscopy (TEM). Since higher bacterial densities were required (5 × 10^8 ^CFU/ml) for electron microscopy, additional colony count assays were performed to determine the antibacterial capacity of CATH-2 at this bacterial density. This yielded a MIC value of 40 μM. In order to determine the concentration dependent effects of CATH-2, *E. coli* were incubated with various concentrations of the peptide (0, 2.5, 5, 10, 40 and 80 μM) for 30 min at 37 °C. Mixtures were fixed with 2% glutaraldehyde (Polysciences, Eppelheim, Germany), 5 mM CaCl_2_, 10 mM MgCl_2_ (both Merck, Darmstadt, Germany) in 0.1 M sodium cacodylate buffer (Sigma-Aldrich) pH 7.4 overnight at 4 °C. In order to observe time dependent effects CATH-2 (0, 5 or 40 μM) was incubated with bacteria (1, 5, 10, 30 min). Killing reactions were stopped by adding the fixative and keeping the cells overnight at 4 °C. After fixation, cells were washed for 3 × 10 min in sodium cacodylate buffer and embedded in 2% low-melting point agarose v/v (Sigma-Aldrich). Subsequently, cells were postfixed with 4% osmium tetroxide (Electron Microscopy Sciences; EMS, Hatfield, USA) and 1.5% K_4_Fe(CN)_6_-3H_2_O (Merck) in distilled water for 2 h at 4 °C and after washing the cells for 5 × 10 min with distilled water, cells were incubated in 0.5% uranylacetate (EMS) for 1 h at 4 °C. After rinsing for 3 × 10 min with distilled water, samples were embedded in Epon and ultrathin sections (50 nm) of each block were prepared on a Leica UCT ultramicrotome (Leica, Vienna, Austria). Lastly, sections were stained with uranyl acetate and lead citrate using the Leica AC20 system (Leica). For all electron microscopy purposes a FEI Tecnai 12 electron microscope (FEI, Eindhoven, The Netherlands) at 80 kV was used.

### Negative staining TEM

Negative staining TEM was used to detect bacterial surface changes after peptide treatment. Bacteria were incubated with various concentrations of CATH-2 (0–40 μM), as previously described for TEM. After fixation, bacteria were rinsed with 0.1 M sodium cacodylate buffer for 3 × 10 min and 100 μl of each bacterial suspension was added on Parafilm. Copper grids (100 mesh) with a Formvar film were carbon coated shortly before use and placed for 5 min on the drop of bacterial suspension. Next, the grids were immersed in the staining solution uranylacetate (0.5%), the excess was removed with filter paper and dried overnight at RT.

### Immunogold TEM

Epon blocks from the TEM experiments were used to determine the localization of the peptide. Sections (50 nm) were mounted on 100 mesh copper Formvar-carbon coated grids, incubated with PBS containing 0.5% fish skin gelatine (Sigma-Aldrich) and 0.1% Bovine Serum Albumin-c^TM^ (AURION, Wageningen, The Netherlands) and immuno-labelled as described before^34^. Sections were incubated with CATH-2 antibody for 1 h at RT, washed extensively with PBS (5 × 2 min) and exposed to protein-A gold (10 nm, Department of Cell Biology, University Medical Centre Utrecht, The Netherlands) for 20 min at RT. Lastly, specimens were stained with 2% uranyloxalicacetate (pH 7; SPI, West Chester, USA) for 5 min at RT and finally embedded in methylcellulose-uranyl acetate (pH 4; 2% methylcellulose [Sigma-Aldrich] and 4% uranylacetate [SPI] in distilled water). In total 50 fields containing 1–3 cells were used for a quantitative analysis of the samples. The labelling densities were determined by counting the intracellular gold particles and gold particles on or nearby the bacterial membrane (all gold particles at a distance of 10 nm or less were considered to belong to the membrane), according to Griffiths^35^. The results from control cells were used to correct for background staining.

### Isothermal titration calorimetry

Interaction between CATH-2 and different compounds (LPS and KDO_2_-Lipid A) was tested using isothermal titration calorimetry (ITC). All ITC experiments were performed on a Low Volume NanoITC (TA instruments - Waters LLC, New Castle, USA). Smooth LPS (LPS-O111:B4) and rough LPS (LPS-K12) were obtained from Invivogen (Toulouse, France) and KDO_2_-Lipid A was obtained from AdipoGen (Liestal, Switzerland). All compounds were diluted in phosphate-buffer (10 mM Na_2_HPO_4_-KH_2_PO_4_, pH 7.4). The syringe was filled with a 50 μl solution of 200 μM CATH-2 and the cell contained 190 μl of a solution of 25 μM LPS or Lipid A. Titrations were incremental with 2 μl injections at 300 seconds intervals. Experiments were performed at 37 °C. Data were analysed with the Nano Analyse software (TA instruments - Waters LLC).

### Competition assays

The inhibition by bacterial membrane compounds (LPS and Lipid A) on the antibacterial activity of CATH-2 was tested. CATH-2 (20 μM final concentration) was pre-incubated with different ratios of LPS-O111:B4 (1:0.25–1:3.25), LPS-K12 (1:0.25–1:2.5) or KDO_2_-Lipid A (1:0.25–1:2.5) for 1 h at 37 °C. Subsequently, the solutions were diluted two-fold in six-steps with distilled water. *E. coli* (2 × 10^6 ^CFU/ml) were added and the mixtures were then incubated for 3 h at 37 °C as for a conventional MIC assay, described in the colony count assay section. After 16 h at 37 °C, the lowest peptide/compound ratio causing inhibition of the antibacterial activity of CATH-2 was determined.

### Statistical analysis

Statistical analysis was performed using a Student’s t-Test. Significant differences were indicated as *(P < 0.05), ***(P < 0.001) or ****(P < 0.0001).

## Additional Information

**How to cite this article**: Schneider, V. A. F. *et al.* Imaging the antimicrobial mechanism(s) of cathelicidin-2. *Sci. Rep.* **6**, 32948; doi: 10.1038/srep32948 (2016).

## Supplementary Material

Supplementary Information

Supplementary Movie S1

Supplementary Movie S2

## Figures and Tables

**Table 1:** MIC values of CATH-2 against *E. coli* strains with rough and smooth LPS.

*E. coli* strain	MIC (μM)
506 (O78K80) (smooth)	10–20
ATCC 25922 (smooth)	10–20
ATCC 4157 (rough/smooth)	5
K12 (rough)	2.5–5
LMC500 (rough)	2.5–5
MC4100 (rough)	5
