# Supplementary material for: Imaging the antimicrobial mechanism(s) of cathelicidin-2
Source: Sci Rep. 2016 Sep 14;6:32948. doi: 10.1038/srep32948 (PMC5021996; doi:10.1038/srep32948)
Supplement: Supplementary Information [file srep32948-s1.pdf]

Supplementary Information:

Imaging the antimicrobial mechanism(s) of cathelicidin-2

Viktoria A. F. Schneider[1], Maarten Coorens[1], Soledad R. Ordonez[1], Johanna L. M. Tjeerdsma-van Bokhoven[1], George Posthuma[2], Albert van Dijk[1], Henk P. Haagsman[1], Edwin J. A. Veldhuizen[1]*

[1]Department of Infectious Diseases and Immunology, Division Molecular Host Defence, Faculty of Veterinary Medicine, Utrecht University, Utrecht, The Netherlands

[2]Department of Cell Biology, Cell Microscopy Core, University Medical Centre Utrecht, Utrecht, The Netherlands

*Corresponding author: e.j.a.veldhuizen@uu.nl

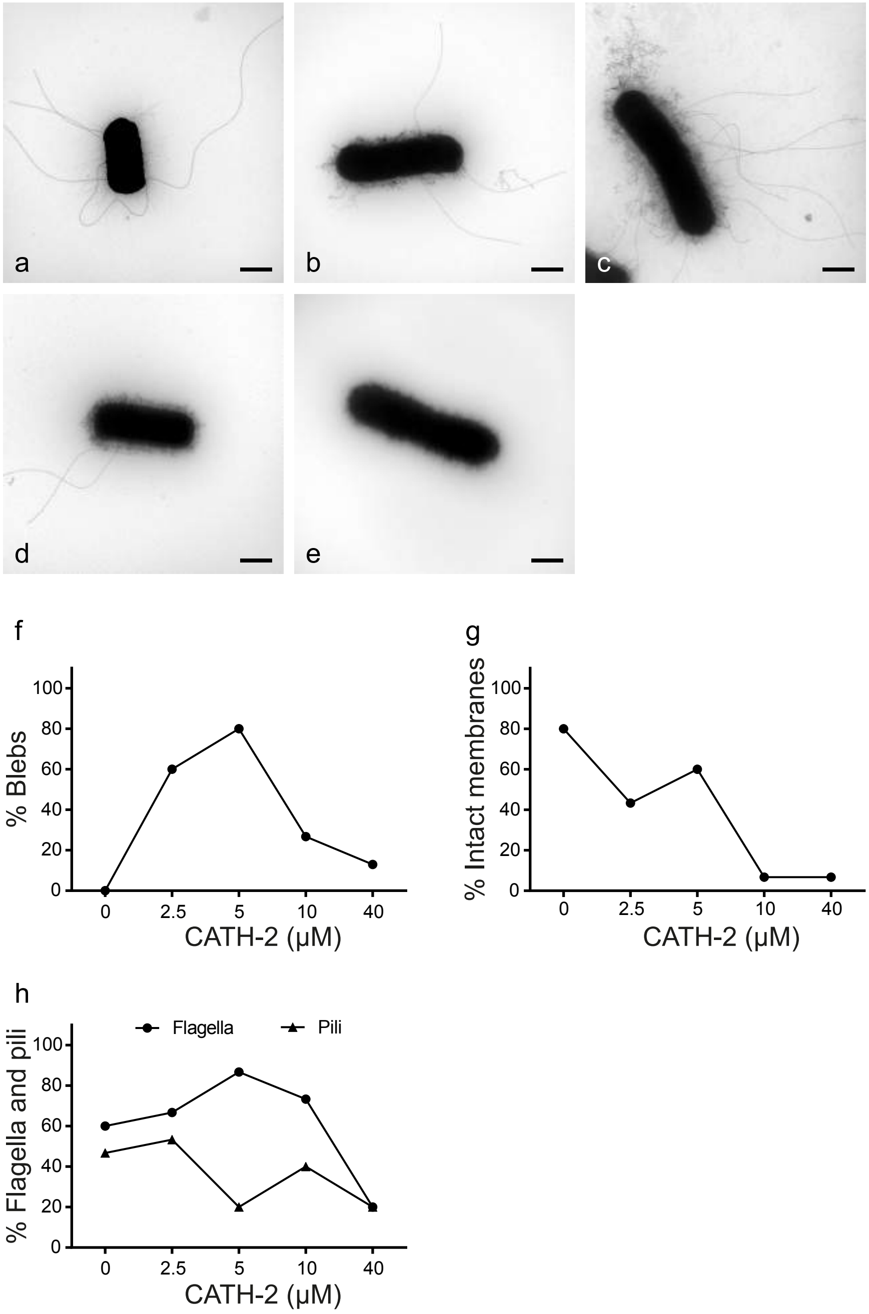

Supplementary Figure S1. CATH-2 induces bleb-formation and at higher concentrations membrane damage as visualized by negative-staining TEM. 0 μM (a), 2.5 μM (b), 5 μM (c), 10 μM (d) and 40 μM (e) were incubated with *E. coli* cells for 30 min. Morphological changes were classified in bleb formation (f), membrane intactness (g) and amount of flagella and pili (h). Bars, 500 nm.

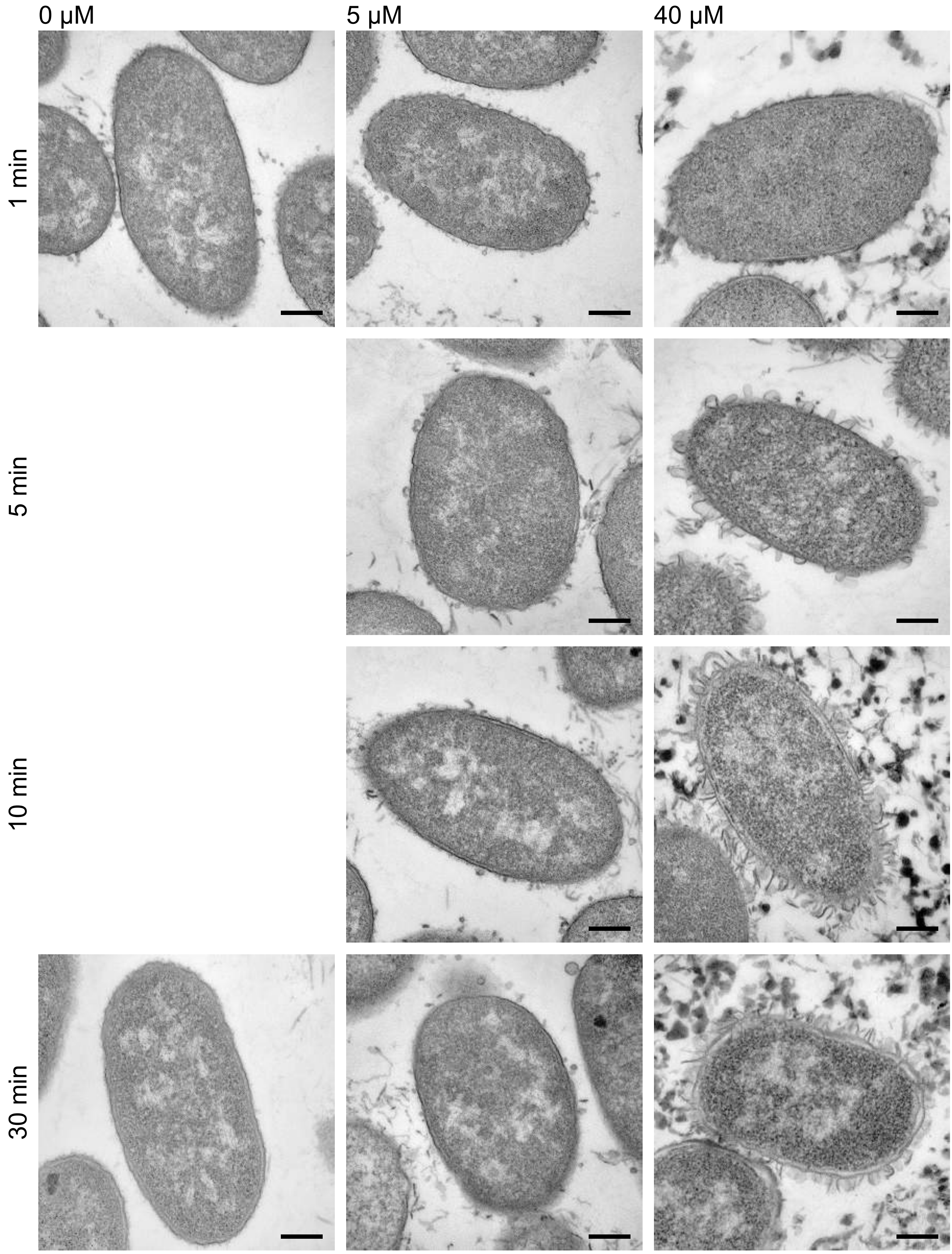

Supplementary Figure S2. CATH-2 causes time dependent morphological changes of *E. coli*. Bacteria were incubated with 5 and 40 μM CATH-2 and reactions were stopped after 1, 5, 10 and 30 min. Representative TEM images are shown for 0 μM, 5 μM and 40 μM. Bars, 200 nm.

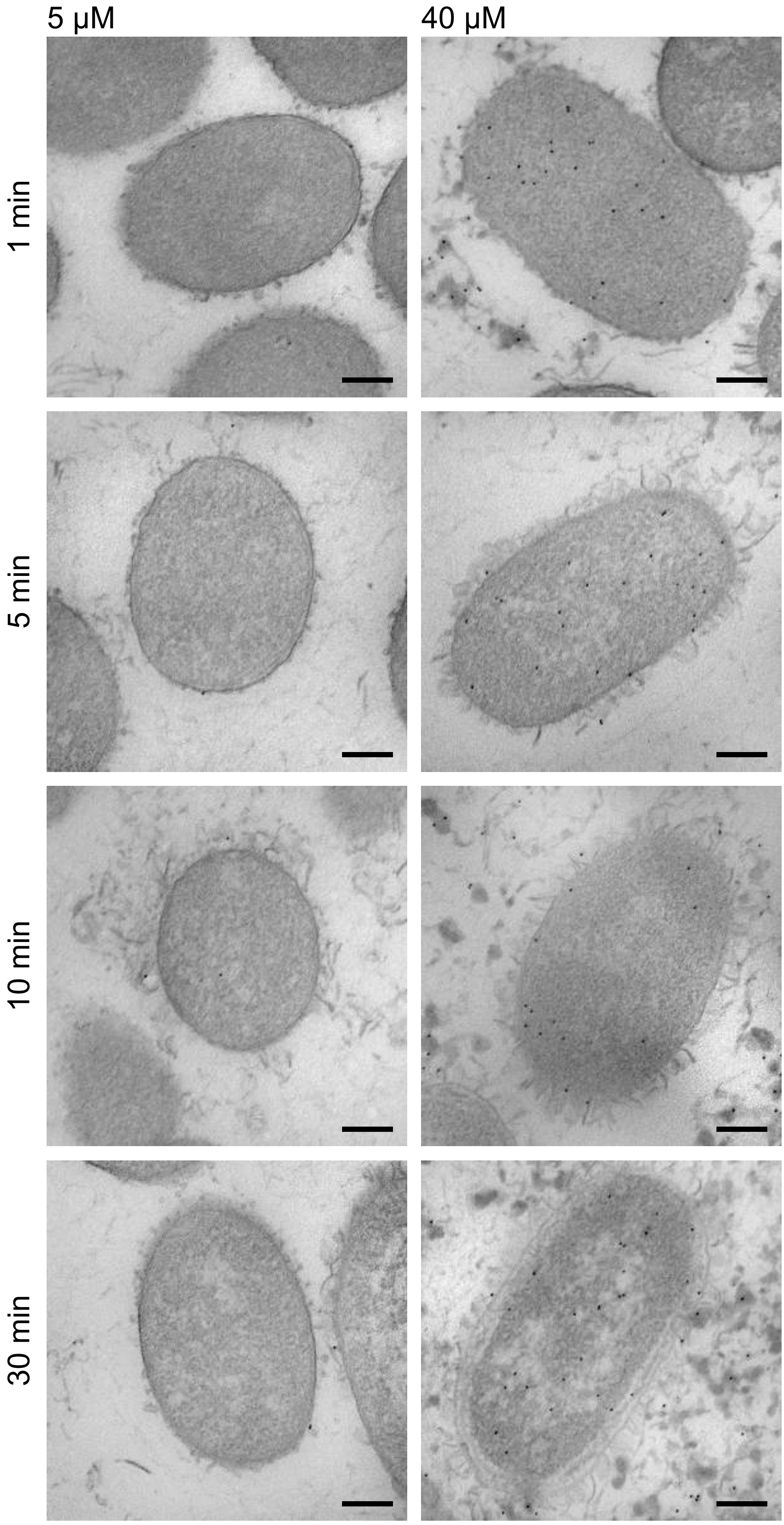

Supplementary Figure S3. Localization of CATH-2 on *E. coli* as a function of time. For each time point a representative TEM image is shown: panel 5 µM and 40 µM both after 1, 5, 10 and 30 min peptide exposure. Bars, 200 nm.

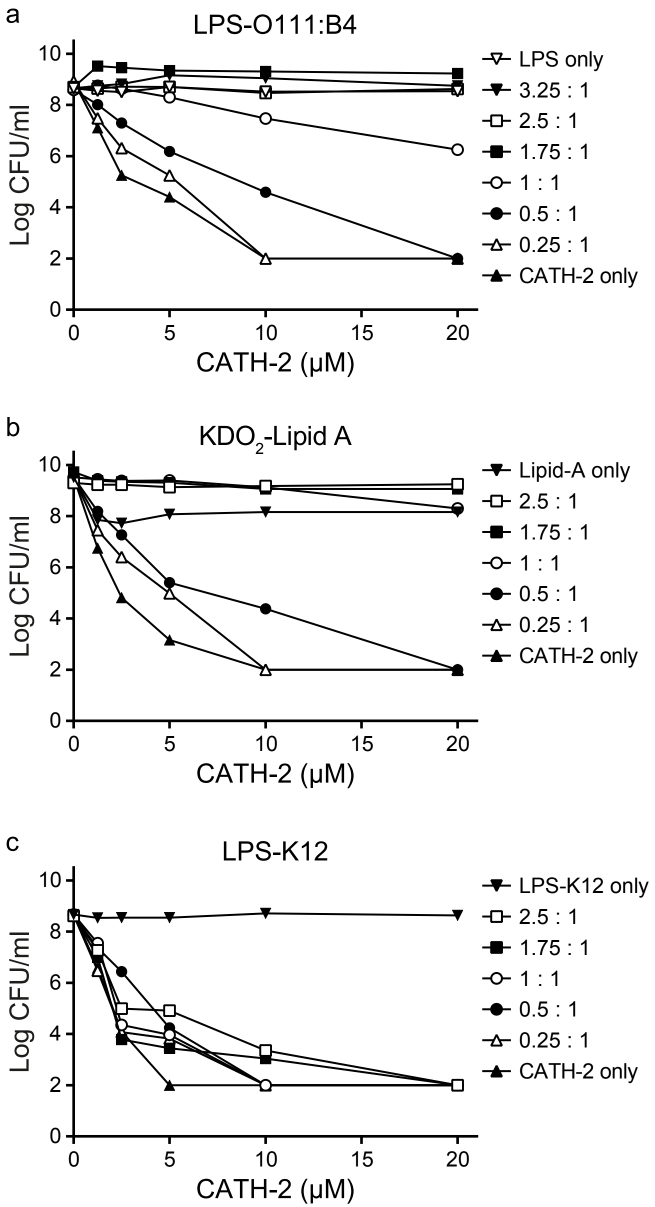

Supplementary Figure S4. LPS and Lipid A inhibit CATH-2 induced killing of *E. coli*. Different ratios of LPS-O111:B4 (a), KDO2-Lipid A (b) and LPS-K12 (c) were pre-incubated with CATH-2 for 1 h at 37 °C. Subsequently mixtures were exposed to *E. coli* for 3 h again at 37 °C and serially diluted and spread plated on TSA plates. Surviving colonies were counted after 16 h. One representative figure per compound is shown (n=3).

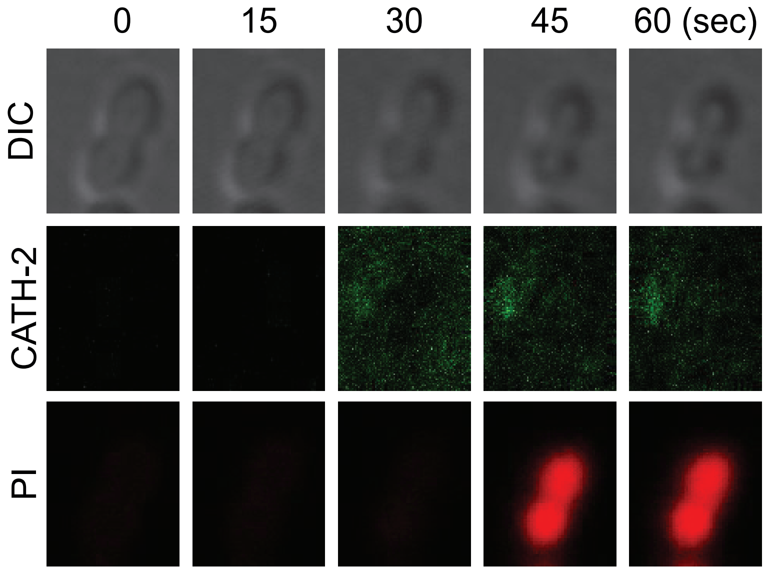

Supplementary Figure 5

Snapshots of *E. coli* K12 show lack CATH-2 binding to the membrane. Time-lapse imaging was performed with 1 μM FITC-CATH-2 (green fluorescence) and 2.5 μM PI (red fluorescence). One representative cell is shown. The first minute of a 6 minute movie is depicted.

Supplementary movie legends

Supplementary Movie S1. CATH-2 binds and permeabilizes the bacterial membrane. Timelapse imaging was performed with 0.9 μM FITC-CATH-2 and 5.1 μM PI. DIC channel, FITC-labelled CATH-2 (green fluorescence), PI (red fluorescence) and custom (merged channels). One representative cell is shown. Bars, 1 μm.

Supplementary Movie S2. CATH-2 permeabilizes multiple *E. coli* cells. Live-imaging with FITC-labelled CATH-2 (0.9 μM) and PI (5.1 μM). Bars, 2 μm.
